# A Novel t(8;14)(q24;q11) Rearranged Human Cell Line as a Model for Mechanistic and Drug Discovery Studies of NOTCH1-Independent Human T-Cell Leukemia

**DOI:** 10.3390/cells7100160

**Published:** 2018-10-09

**Authors:** Valeria Tosello, Gloria Milani, Annalisa Martines, Nadia Macri, Wouder Van Loocke, Filip Matthijssens, Barbara Buldini, Sonia Minuzzo, Deborah Bongiovanni, Richard Fabian Schumacher, Alberto Amadori, Pieter Van Vlierberghe, Erich Piovan

**Affiliations:** 1 UOC Immunologia e Diagnostica Molecolare Oncologica, Istituto Oncologico Veneto—IRCCS, Padova 35128, Italy; valeria.tosello@iov.veneto.it (V.T.); annalisa.martines@iov.veneto.it (A.M.); nadia.macri@iov.veneto.it (N.M.); albido@unipd.it (A.A.); 2 Department of Biomolecular Medicine, Ghent University, Ghent 9000, Belgium; gloria.milani@gmail.com (G.M.); Wouter.VanLoocke@UGent.be (W.V.L.); Filip.Matthijssens@Ugent.be (F.M.); pieter.vanvlierberghe@ugent.be (P.V.V.); 3 Cancer Research Institute Ghent (CRIG), Ghent 9000, Belgium; 4 Dipartimento di Salute della Donna e del Bambino, Università degli Studi di Padova, Padova 35128, Italy; barbara.buldini@unipd.it; 5 Dipartimento di Scienze Chirurgiche, Oncologiche e Gastroenterologiche, Universita’ degli Studi di Padova, Padova 35128, Italy; soniaanna.minuzzo@unipd.it (S.M.); deborah.bongiovanni@studenti.unipd.it (D.B.); 6 Pediatric Oncology Unit of Spedali Civili di Brescia, Brescia 25123, Italy; fabian.schumacher@unibs.it

**Keywords:** MYC-translocated leukemia, T-lineage acute lymphoblastic leukemia, NOTCH1-independent, super-enhancers, BRD4 inhibition, targeted therapy

## Abstract

*MYC*-translocated T-lineage acute lymphoblastic leukemia (T-ALL) is a rare subgroup of T-ALL associated with *CDKN2A/B* deletions, *PTEN* inactivation, and absence of *NOTCH1* or *FBXW7* mutations. This subtype of T-ALL has been associated with induction failure and aggressive disease. Identification of drug targets and mechanistic insights for this disease are still limited. Here, we established a human NOTCH1-independent *MYC*-translocated T-ALL cell line that maintains the genetic and phenotypic characteristics of the parental leukemic clone at diagnosis. The University of Padua T-cell acute lymphoblastic leukemia 13 (UP-ALL13) cell line has all the main features of the above described *MYC*-translocated T-ALL. Interestingly, UP-ALL13 was found to harbor a heterozygous R882H *DNMT3A* mutation typically found in myeloid leukemia. Chromatin immunoprecipitation coupled with high-throughput sequencing for histone H3 lysine 27 (H3K27) acetylation revealed numerous putative super-enhancers near key transcription factors, including MYC, MYB, and LEF1. Marked cytotoxicity was found following bromodomain-containing protein 4 (BRD4) inhibition with AZD5153, suggesting a strict dependency of this particular subtype of T-ALL on the activity of super-enhancers. Altogether, this cell line may be a useful model system for dissecting the signaling pathways implicated in NOTCH1-independent T-ALL and for the screening of targeted anti-leukemia agents specific for this T-ALL subgroup.

## 1. Introduction

T-cell acute lymphoblastic leukemia (T-ALL) represents approximately 15% of pediatric and 25% of adult ALL cases and is characterized by a high prevalence of activating mutations in the *NOTCH1* gene [1]. T-ALL is a fairly heterogeneous disease which includes several major subgroups associated with specific chromosomal rearrangements and is defined by characteristic gene expression signatures such as TAL-LMO, TLX1/TLX3, or HOX-like [2,3,4]. Interestingly, the presence of *NOTCH1* mutations appear to be associated with a favorable therapeutic response, while NOTCH1-independent T-ALL cases have a less favorable prognosis [5]. However, conflicting results have been reported on the prognostic impact of *NOTCH1* activating mutations, possibly due to differences in therapy intensification [6]. An understanding of the genetic/molecular pathways implicated in and sustaining NOTCH1-independent T-ALLs is required to identify novel therapies. An emerging group of NOTCH1-independent TAL/LMO-positive leukemias harboring *MYC* translocations (constituting around 1–6% of adult and childhood T-ALL cases) has been recently described [7,8]. This rare subgroup frequently presents with aggressive disease and poor response to standard therapy. Currently, a limited number of cell lines are available that are *NOTCH1* or *NOTCH3* wild-type (wt), such as MOLT-16 [9]. Interestingly, MOLT-16 [10] is also characterized by t(8:14)(q24;q11)/*TCRAD*-*MYC* translocation, *SIL-TAL1* and *LMO2* translocations as primary alterations, and *CDKN2A/B* deletions and *PTEN* deletions or mutations as additional abnormalities. The genetic profile of this cell line and leukemia cases containing t(8:14)(q24;q11) leading to MYC overexpression with *NOTCH1*wt/*FBXW7*wt/*PTEN* mutation or deletion resembles that of a recently described Notch1-independent mouse leukemia model arising following conditional *Pten* deletion [11]. This profile is also similar to a NOTCH1-independent/MYC-mediated T-ALL subset, where concurrent PTEN down-regulation/inactivation contributes to MYC over-expression [12]. Given the recent limitations reported with established cell lines, including multiple transformations and derivations, misidentification, and cross-contamination with other cell line(s) [13], it would be desirable to test and develop anti-cancer drugs using well-characterized cell lines that preserve patterns of responsiveness to micro-environmental stimuli and maintain the integrity of the signaling pathways engaged by these stimuli. In contrast to primary leukemia cells, which undergo spontaneous apoptosis in vitro and whose viability can be rescued by cytokine cocktails [14,15] or stromal cells [16] (suggesting that normally in vivo micro-environmental cues are important for sustaining their growth and survival), available T-ALL cell lines have lost this trait. This may be particularly relevant for NOTCH1-independent T-ALL cell lines where only few examples exist and have been extensively cultured in vitro. As part of our efforts to develop better tools for understanding the role of MYC activation and PTEN loss-of-function in NOTCH1-independent T-ALL, we established a new cell line named University of Padua T-cell acute lymphoblastic leukemia 13 (UP-ALL13) harboring t(8:14)(q24;q11) with co-occurring abnormalities including deletions/alterations in *CDKN2A/B*, *SIL-TAL1*, *PTEN*, and *LMO2*. In addition, sequencing of UP-ALL13 revealed additional mutations in a limited number of genes, including a heterozygous R882H mutation affecting DNA methyltransferase enzyme 3A (*DNMT3A*) that is typically found in myeloid leukemia [17]. In this cell line, we also describe genes associated with putative super-enhancers (defined by exceedingly high levels of histone H3 lysine 27 acetylation (H3K27ac)). Finally, we evaluated the sensitivity of UP-ALL13 to therapeutic agents either currently used for the treatment of T-ALL or novel targeted therapies which could be effective in this model of NOTCH1-independent leukemia.

## 2. Materials and Methods

Patient material: Primary leukemia cells were obtained from the peripheral blood (PB) of a 5-year-old male patient at diagnosis. Informed consent and approval by the Azienda Ospedaliera di Padova Review Board were obtained according to general guidelines, conforming with the Declaration of Helsinki.

Establishment and in vitro maintenance of the UP-ALL13 cell line: Procedures involving animals and their care conformed with institutional guidelines that comply with national and international laws and policies (European Economic Community (EEC) Council Directive 86/609, OJ L 358, 12 December 1987) and were authorized by the local ethical committee on animal welfare. Primary T-ALL cells (10 × 10^6^) from PB were injected in vivo via intravenous injection (IV) into 6–8 week old female Nonobese diabetic/severe combined immunodeficiency interleukin 2 receptor gamma chain null (NOD SCID IL2Rγ^null^) (NSG) immunodeficient mice. Mice were euthanized upon development of signs of leukemia, at which point PB, spleen, and bone marrow (BM) were collected and analyzed for the presence of human leukemic cells (as judged by cluster of differentiation 45 (CD45) and/or CD7 expression). Authenticated human leukemia cells isolated from the spleen were then re-passaged in the mice. After approximately five passages in the mice, in vitro culturing of leukemia cells was attempted by seeding cells in 24-well plates at 2 and 4 × 10^6^/mL in Minimum Essential Medium (MEM-alpha) supplemented with 10% fetal bovine serum (FBS), 10% human heat-inactivated AB serum, antibiotics, ultra-glutamine, and 10 ng/ml recombinant Interleukin-7 (IL-7) (PeproTech, London, United Kingdom). This strategy was used for the initial month of in vitro culture; subsequently, leukemic cells were cultured in Roswell Park Memorial Institute (RPMI)-1640 medium supplemented with 20% FBS (RPMI–20%FBS) at a density of 2 × 10^6^/mL. Medium was changed periodically, and cells were passaged every 5–6 days. UP-ALL13 cells were maintained in continuous culture in RPMI–20% FBS for more than 9 months.

Immunophenotype: Primary leukemia cells were phenotyped at the moment of diagnosis by using standard protocols [18]. Flow cytometric analysis of freshly isolated xenograft cells (passage ≈ 10) and UP-ALL13 cells (≈8 months of in vitro culture) was performed using fluorochrome-conjugated monoclonal antibodies (mAbs) (Table 1) obtained from BD Pharmingen (San Diego, CA, USA) and Beckman-Coulter (Miami, FL, USA). Samples were analyzed using the BD FACSCanto™ II (Beckman-Dickinson, Roswell Park Memorial Institute, NY, USA) flow cytometer.

Clonality assessment, DNA fingerprinting, and cytogenetic and fluorescence in situ hybridization analysis: T-cell receptor (TCR) gene rearrangements were analyzed in the primary leukemia cells at diagnosis and in cells obtained from the xenograft in order to check if clonality was maintained after passage into NSG mice. Genomic DNA was isolated from primary leukemia cells at diagnosis and xenograft cells, and PCR analysis was performed to detect *TCRD*, *TCRG*, and *TCRB* rearrangements using methods and primers previously described [19,20]. Clonal gene rearrangements, identified by homo/heteroduplex analysis, were sequenced by a dye-terminator cycle sequencing kit on an ABI Prism 310 apparatus (Life Technologies, Carlsbad, CA, USA) [21]. The genetic identity of the derived cell line with respect to the original primary leukemia cells from the patient was confirmed by analyzing several loci of short tandem repeats (STRs) using a commercial kit (PowerPlex 16 HS System, Madison, WI, USA). Metaphase chromosome preparations were obtained from the UP-ALL13 cell line after overnight exposure to 100 ng/mL colcemid (KaryoMAX Colcemid solution, Life Technologies, Carlsbad, CA, USA). G-banding was performed with Wright Stain (Sigma Aldrich, St. Louis, MO, USA) and the karyotype was described following International System for Human Cytogenetic Nomenclature (ISCN) 2016 nomenclature, after the analysis of 25 metaphases with IKAROS software (Metasystems, Altlussheim, Germany). Fluorescence in situ hybridization (FISH) was performed by standard method with a break-apart probe for MYC (Zytolight SPEC MYC dual break-apart probe, ZytoVision, Bremerhaven, Germany). Hybridization signals were scored on at least 10 metaphases and 100 interphase nuclei using ISIS software (Metasystems) and an AxioImager Z2 microscope (Zeiss, Jena, Germany) equipped with appropriate filters.

Proliferation, apoptosis, and cell cycle analysis after treatment with signaling-specific inhibitors and chemotherapeutic drugs: T-ALL cell lines were purchased from the Deutsche Sammlung von Mikroorganismen und Zellkulturen (DSMZ) repository (Braunschweig, Germany) and cultured at 37 °C (5% CO_2_) in RPMI–10% FBS. All cell lines were periodically authenticated by STR profiling and tested for *Mycoplasma* contamination. We analyzed cell viability in UP-ALL13, *NOTCH1* mutant T-ALL cell lines (DND41, CUTLL1) and established t(8;14)(q24:q11)-translocated T-ALL cell lines (MOLT-16, SKW-3/KE-37) via the bioluminescent method Vialight plus (Lonza, Basel, Switzerland) after the indicated time points. In detail, duplicate cultures of UP-ALL13 cells (5 × 10^5^) or T-ALL cell lines (3 × 10^5^) were seeded in 24-well flat-bottomed plates and treated with increasing doses of various compounds: EPZ5676, AZD5153, JQ1, and EPZ004777 (Selleck Chemicals LLC, Houston, TX, USA), etoposide and dexamethasone (Sigma-Aldrich), and dibenzazepine (DBZ; Syncom, Groningen, the Netherlands). All drug experiments were repeated at least twice, with similar results. We analyzed apoptosis by flow cytometry (FACS) after staining with Annexin V- Fluorescein-5-Isothiocyanate (FITC) (Roche, Burgess Hill, UK) and SYTOX Red dead cell stain (Invitrogen, Paisley, UK). Cell cycle distribution was performed by assessing DNA content using propidium iodide (PI; Sigma). The samples were collected on a FACSCalibur (BD Biosciences, Milan, Italy) using Cell Quest software (BD Biosciences) and analyzed with FlowJo (FLOWJO LLC, Ashland, OR, USA).

Array comparative genomic hybridization (aCGH): Copy number alterations were profiled in cells obtained from the patient xenograft and UP-ALL13 cell line using the 180K custom-designed oligonucleotide array platform (Agilent SurePrint G3 Human CGH microarrays, G4449A, design ID: 022060). Genomic DNA from samples and standard controls was labeled using random prime labeling with Cy3 and Cy5 dyes (Perkin Elmer, Waltham, MA, USA). Hybridization was performed according to the manufacturer’s instructions (Agilent Technologies, Santa Clara, CA, USA). Data were analyzed using the Comprehensive Platform for the analysis and visualization of Structural Genomic Variation (ViVar) software [22] arrayCGHbase tool.

Whole exome sequencing and mutational analysis: Whole exome sequencing (WES) was performed using DNA extracted from PB patient mononuclear cell pellets which was subjected to Sure Select capture technology (Human All Exome V6 Kit, Agilent) and paired-end sequencing on an Hiseq3000 device (Illumina Inc., San Diego, CA, USA). Alignment with the reference genome was performed using a Burrows–Wheeler Aligner (BWA) and somatic variant calling was executed by MuTect [23] and Strelka [24]. 

Mutations detected by WES of the genes: *ABCA4*, *DNMT3A*, *FADS6*, *FGFR3*, *GNB2*, *GPR39*, *IFIT1B*, *LRRC74A*, *NBPF10*, *PCDHB6*, *PMM1*, *SCRIB*, *SPATA31D3*, *VCX*, and *LCE1E* were independently amplified and subjected to Sanger sequencing. Mutational analysis was performed on two independent PCR products containing the putative mutation found by WES and evaluated using DNAdynamo tool. Primers of sequenced genes with allelic frequency (AF) > 0.22 are listed in the supplement (Supplementary Table S1).

Immunoblot analysis: Total cell lysates were prepared using RadioImmunoprecipitation Assay (RIPA) lysis buffer supplemented with phosphatase inhibitor cocktail set I and II (Sigma) and protease inhibitor cocktail tablets (Roche) and normalized for protein concentration using the Bicinchoninic Acid (BCA) method (Pierce, Pero, Italy). For Western blotting, protein samples were separated on 4–12% gradient Tris–glycine SDS-PAGE (Invitrogen) and transferred to PVDF membrane (Millipore, Burlington, MA, USA). Antibodies against Protein Tyrosine Phosphatase Receptor Type C (PTPRC/CD45) (H-230, sc-25590), c-myc (9E10, sc-40), and p53 (DO-1, sc-126) were from Santa Cruz Biotechnology (Dallas, Texas, USA); antibodies recognizing CXCR4 (ab124824) and MYB (ab117635) were from Abcam (Cambridge, UK); and antibodies recognizing LEF1 (C12A5, #2230), cleaved caspase 3 (#9661), and β-actin (D6A8, #8457) were from Cell Signaling Technologies (Danvers, MA, USA).

H3K27ac chromatin immunoprecipitation sequencing (ChIP-seq): UP-ALL13 cells (2 × 10^7^) were cross-linked with methanol-free formaldehyde (1% final concentration) at room temperature for 7 min and the cross-linking reaction was quenched with glycine (125 mM final concentration, Sigma-Aldrich). Nuclei were isolated and chromatin was purified by chemical lysis (truChIP Chromatin Shearing Reagent KIT, Covaris, Woburn, MA, USA) and processed as previously described [8].

Statistical analysis: We performed statistical analysis by Student’s *t*-test and Mann-Whitney *U* test where appropriate. All statistical tests were two-sided, and *p* < 0.05 was considered statistically significant. Results are shown as mean of quadruplicate wells. Error bars represent ± standard deviation. Survival in animal experiments was represented with Kaplan–Meier curves (GraphPad Prism Software, San Diego, CA, USA).

## 3. Results

### 3.1. Establishment of a New NOTCH1-Independent T-ALL Cell Line that Engrafts in Immune-Deficient Mice

The considerable rate of spontaneous apoptosis and lack of significant spontaneous in vitro expansion of leukemic T cells prompted us to use a different strategy to establish novel NOTCH1-independent T-ALL cell lines. In fact, to circumvent the obstacles encountered following in vitro culture, we established a new cell line through serial passages in the permissive microenvironment of NSG mice. Lymphoblast cells from the PB of a 5-year-old boy at the time of diagnosis were injected into NSG mice. The main clinical features of the leukemia patient are summarized in supplemental Table S2. In all animals initially injected (*n* = 3), the leukemic cells engrafted with leukemia, infiltrating numerous organs including the bone marrow and spleen with a time to leukemia of approximately 35 days. No evidence of thymus enlargement or mediastinal mass was observed. Leukemic cells were recovered from the spleen of diseased animals and injected into secondary recipient animals. Prior to each in vivo passage, authenticity of the leukemic cells with respect to the original patient material was evaluated through TCR rearrangement and DNA microsatellite fingerprints (STR). After the fifth in vivo passage, in vitro culturing of leukemic cells from diseased animals was attempted. After numerous attempts, a cell line was obtained that grew continuously in culture. This cell line was named UP-ALL13 and was in continuous culture for over 9 months. UP-ALL13 cells show a doubling time of 60–72 h and can be frozen and thawed successfully. Microsatellite fingerprinting results from UP-ALL13 and primary leukemia cells used to establish this cell line demonstrated that they share a unique DNA fingerprint. TCR rearrangements and STR results for the UP-ALL13 cell line, xenograft, and primary sample are provided in the supplemental material (Table S3).

**Figure 1:**
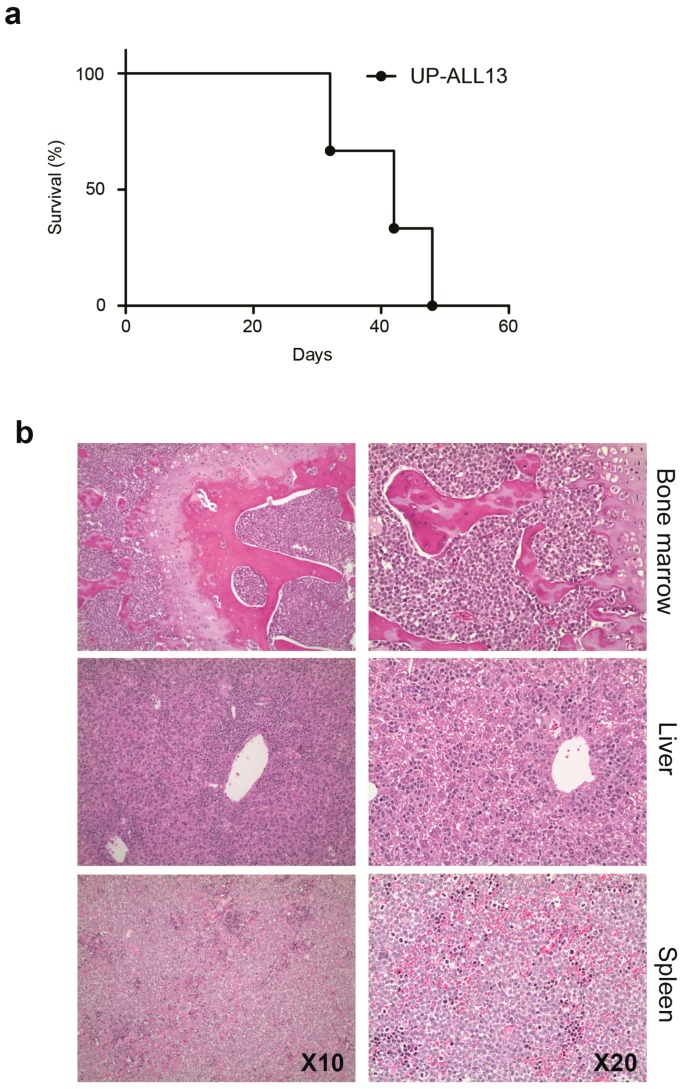
The University of Padua T-cell acute lymphoblastic leukemia 13 (UP-ALL13) engrafts into Nonobese diabetic/severe combined immunodeficiency (NOD/SCID) interleukin 2 receptor gamma chain null (IL2Rγnull) (NOD SCID IL2Rγ^null^; NSG) mice and induces leukemia. (**a**) Survival curve for NSG mice engrafted intravenously with UP-ALL13 cells (5 × 10^6^ cells; *n* = 3). (**b**) Histology of representative infiltrated organs of moribund mice (hematoxylin and eosin staining; H&E). Magnification: ×10 (left panels); ×20 (right panels).

Animal models to study molecular mechanisms as well as drug efficacy of NOTCH1-independent T-ALL in vivo are desirable to further enhance mechanistic studies. To determine tumorigenicity, UP-ALL13 cells were transplanted IV into NSG mice (5–10 × 10^6^ viable cells/mouse). In all animals tested (*n* = 3), UP-ALL13 cells engrafted and determined leukemia disseminating to multiple organs. Animals developed disease and were euthanized at 37.7 ± 9 days after cell injection (range, 32–48 days; Figure 1a). Histological analysis disclosed the involvement of BM and lymphoid organs such as the spleen and liver in all animals (Figure 1b). Involvement of kidneys was also noted (not shown), with no evidence of thymus enlargement or mediastinal mass. Importantly, recovered leukemic cells from diseased mice maintained the capacity to grow when cultured in vitro and maintained their genetic identity (data not shown).

### 3.2. UP-ALL13 Is a New T-ALL Cell Line with t(8;14)-Translocation Involving the MYC Proto-Oncogene and Presenting a Heterozygous R882H DNMT3A Mutation

To evaluate whether UP-ALL13 share the patient’s primary leukemia cell phenotype, immunophenotypic and clonality analyses were executed in uncultured primary leukemia cells, xenograft cells, and UP-ALL13 cells cultured in vitro. Immunophenotypic analysis of the UP-ALL13 cell line and primary sample disclosed an overlapping phenotype with expression of CD7, CD2, CD5, and surface CD3 (sCD3), and lack of CD1a, B-cell (CD19), or natural killer (CD16/CD56) cell surface markers (Table 1). Interestingly, some myelomonocytic lineage markers (CD13 and possibly CD15) were partially expressed in the UP-ALL13 cell line compared to the parental cells. Altogether, this pattern of expression is consistent with a mature thymocyte phenotype of both primary sample and derived T-ALL cell line.

**Figure 2:**
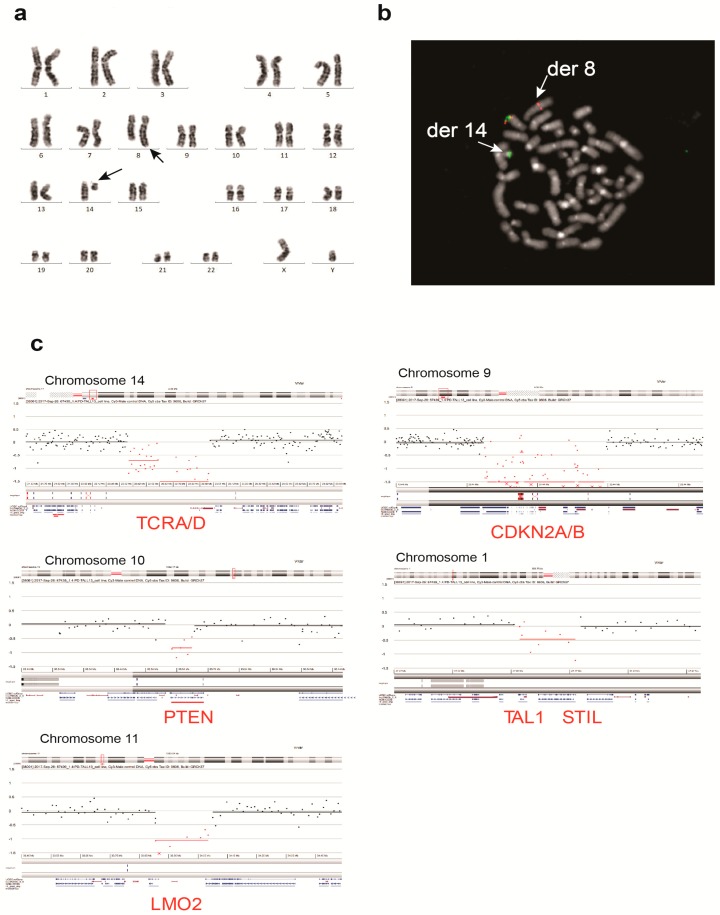
Cytogenetic and molecular characterization of the UP-ALL13 cell line. (**a**) Cytogenetic analysis described a t(8;14)(q24;q11) translocation as a single clonal chromosomal abnormality (arrows). (**b**) Fluorescence in situ hybridization (FISH) analysis performed on metaphases using a break-apart probe for MYC confirmed MYC rearrangement on t(8;14)(q24;q11) translocation by demonstrating the presence of the 5’MYC probe on the derivative chromosome 8 (der 8) and the translocation of the 3’ MYC probe on the derivative chromosome 14 (der 14). (**c**) Array comparative genomic hybridization (CGH) results of UP-ALL13 cells showing deletions involving *TCRA/D*, *CDKN2A/B*, *PTEN*, *TAL1/STIL*, and *LMO2* loci.

Cytogenetic analysis of the UP-ALL13 cell line showed a diploid male karyotype of 46 chromosomes with a single chromosomal translocation t(8;14)(q24;q11): 46, XY, t(8;14)(q24;q11) (Figure 2a). This recurrent chromosomal translocation typically involves the *MYC* proto-oncogene (mapping on 8q24) and the *TCRA/D* locus in chromosome 14q11, and it is found in approximately 1–6% of adult and pediatric T-ALL cases [7]. The involvement of the *MYC* proto-oncogene was further confirmed by FISH on metaphases with a break-apart probe for MYC (Figure 2b). Targeted locus amplification (TLA) [25] further corroborated the above results (data not shown). aCGH on the primograft (not shown) and the established UP-ALL13 cell line (Figure 2c), confirmed the *TCR A/D* deletion and revealed a limited number of additional alterations including bi-allelic deletions of *CDKN2A/B* and mono-allelic deletions of *PTEN* together with *STIL/TAL1* and *LMO2* alterations. On the other hand, no mutations were found by targeted Sanger sequencing in other genes commonly altered in T-ALL, such as *FBXW7*, *PHF6*, *WT1*, and most notably *NOTCH1* (data not shown). In agreement with *NOTCH1* sequencing data, treatment with a gamma secretase inhibitor (dibenzazepine; DBZ) for a prolonged period of time (6–7 days) did not significantly impact on proliferation and did not induce cell cycle arrest (Supplementary Figure S1), suggesting independence from NOTCH1 signaling for leukemia maintenance. Thus, the genetic profile of our leukemia cell line with t(8;14)(q24;q11) translocation and *NOTCH1*wt/*FBXW7*wt/*PTEN* deletion is compatible with a recently identified NOTCH1-independent/MYC-mediated T-ALL subset [12].

**Figure 3:**
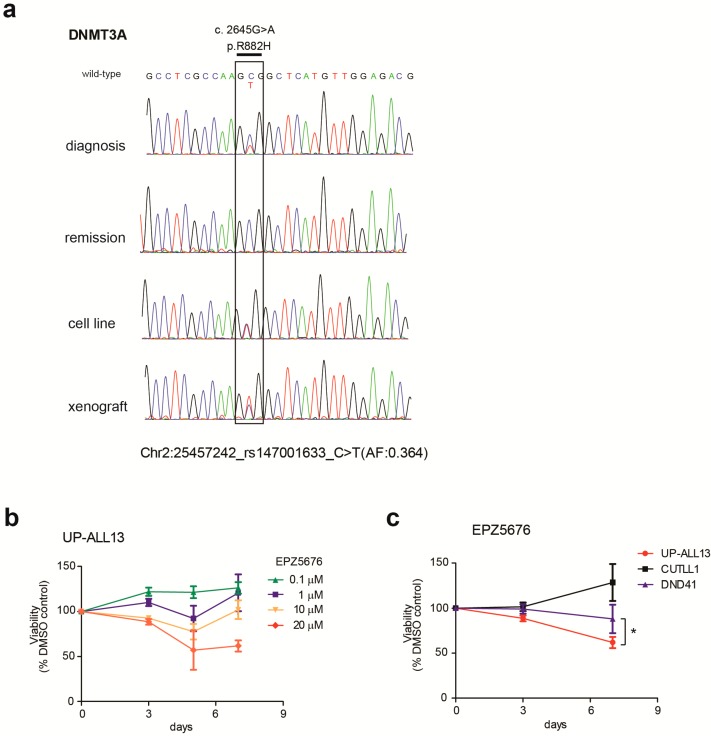
Mutational analysis of the *DNMT3A* mutational hotspot (R882) and response of the UP-ALL13 cell to the DOT1L inhibitor EPZ5676. (**a**) Sanger sequencing of UP-ALL13 cells validates the point mutation (c.2645G > A; pR882H) affecting the *DNMT3A* gene, as identified by whole exome sequencing (WES) in the patient leukemia cells at diagnosis. (**b**) Cell viability of UP-ALL13 cells treated for up to 7 days with different doses of the DOT1L inhibitor EPZ5676. Viability is shown as a percentage of the Dimethyl Sulfoxide (DMSO) control cells at each time point. Error bars represent ± standard deviation of quadruplicate wells. (**c**) Cell viability of UP-ALL13, CUTLL1, and DND41 cells treated for 3 and 7 days with a dose of 20 μM of EPZ5676. Viability is shown as a percentage of the DMSO control cells at each time point. Error bars represent ± standard deviation of quadruplicate wells. *, *p* < 0.05.

To further investigate the genetic profile of our NOTCH1-independent T-ALL, we performed WES of the parental diagnosis (and remission) DNA sample. This analysis allowed us to detect mutations in additional genes (*ABCA4*, *DNMT3A*, *FADS6*, *FGFR3*, *GNB2*, *GPR39*, *IFIT1B*, *LRRC74A*, *NBPF10*, *PCDHB6*, *PMM1*, *SCRIB*, *SPATA31D3*, *VCX*, and *LCE1E*). Of these genes, we were able to validate by Sanger sequencing mutations affecting *DNMT3A*, *LRRC74A*, *PCDHB6*, *PMM1*, *ABCA4*, and *SPATA31D3* which had an AF > 0.22 (Figure 3a and Supplementary Figure S2). All these additional mutations were replicated in the DNA obtained from primo-xenograft and the UP-ALL13 cell line, further demonstrating their clonal derivation from the patients’ leukemic cells. Particularly interesting was the finding of the heterozygous R882H mutation affecting *DNMT3A*, which is a mutational hotspot in acute myeloid leukemia (AML) and early T-cell precursor (ETP) adult T-ALL, but is rarely encountered in pediatric T-ALL [26].

Recently, murine hematopoietic stem cells (HSCs) in which *Dnmt3a* had been conditionally deleted were shown to overexpress the histone 3 lysine 79 (H3K79) methyltransferase *Dot1l*, leading to increased H3K79 DNA methylation [27]. Pharmacological inhibition of DOT1L by EPZ5676 or EPZ004777 leads to a significant anti-leukemic effect in vitro and in vivo in *DNMT3A*-mutant AML primary samples and cell lines [27]. Since our T-ALL cell line (UP-ALL13) also harbors a R882 *DNMT3A* mutant, we evaluated in vitro the therapeutic efficacy of DOT1L inhibition by EPZ5676 [28]. We found only a modest dose- and time-dependent reduction in cell viability (Figure 3b). Indeed, only prolonged exposure to high doses of EPZ5676 (20 µM) for 7 days significantly impacted cell survival, as compared to two T-ALL cell lines (CUTLL1, DND41) not known to harbor DNMT3A mutants (Figure 3c). A similar effect was seen using another DOT1L inhibitor such as EPZ004777 (data not shown). This finding may be due to the different mechanism of action of the *DNMT3A* mutation in T-ALL compared to AML [27].

### 3.3. A Super-Enhancer Portrait in the NOTCH1-Independent T-ALL Cell Line UP-ALL13

**Figure 4:**
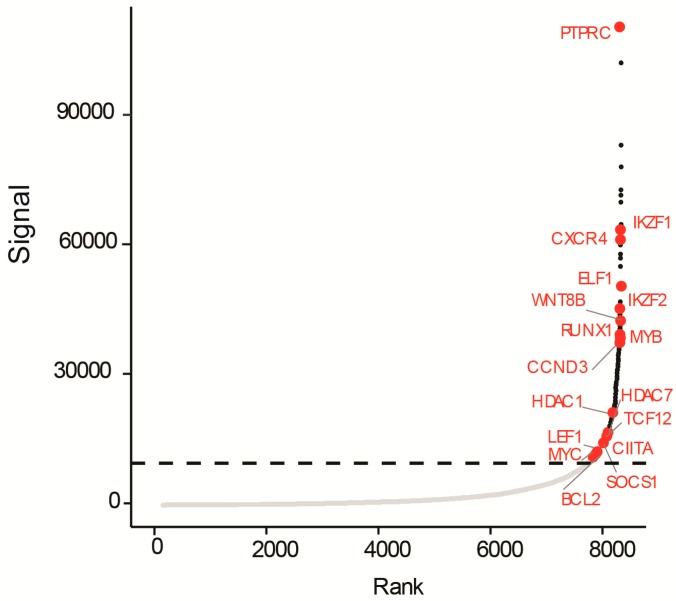
Histone H3 lysine 27 acetylation (H3K27ac) chromatin immunoprecipitation (ChIP) sequencing identifies putative super-enhancers in UP-ALL13 cells. Hockey-stick plot representing the normalized rank and signal of H3K27ac peaks in t(8;14)(q24;q11)-positive UP-ALL13 cells. Representative super-enhancer-associated genes are shown in red.

Not much is known regarding the enhancer elements which are important for maintaining NOTCH1-independent T-ALL. In order to identify active enhancers, promoters, and super-enhancers [29], we performed chromatin immunoprecipitation coupled to high-throughput sequencing (ChIP-seq) with an antibody against the enhancer-associated histone modification H3K27ac on UP-ALL13 cells. We found numerous regions with high levels of H3K27ac, with exceptionally high levels of H3K27ac in a relatively small set of large enhancer regions (possibly super-enhancers). Enhancers tend to loop to and associate with adjacent genes in order to activate transcription [30,31,32]. Most of these interactions occur within a distance of ≈50 kb of the enhancer [33]. Using a simple proximity rule, we assigned transcriptionally active genes to super-enhancers within a 50-kb window [34]. This procedure allowed us to identify numerous genes associated with super-enhancers, including various genes implicated in T-ALL and cancer pathogenesis in general, such as *PTPRC* (CD45), *IKZF1* (Ikaros), *CXCR4*, *ELF-1*, *IKZF2* (Helios), *WNT8B*, *CCND3*, *RUNX1*, *HDAC1* and *7*, *SOCS1*, *CIITA*, *TCF12*, *LEF1*, *MYB*, *BCL2*, and *MYC* (Figure 4). The identification of key oncogene drivers regulated by super-enhancers in UP-ALL13 could help in determining the oncogenic drivers behind NOTCH1-independent T-ALL, which could cause a predisposition to disproportionate sensitivity to loss of bromodomain and extraterminal (BET) chromatin reader proteins such as the bromodomain-containing protein 4 (BRD4) cofactor.

### 3.4. UP-ALL13 Is Highly Sensitive to the Bromodomain-Containing Protein 4 (BRD4) Inhibitor AZD5153 and Glucocorticoids

**Figure 5:**
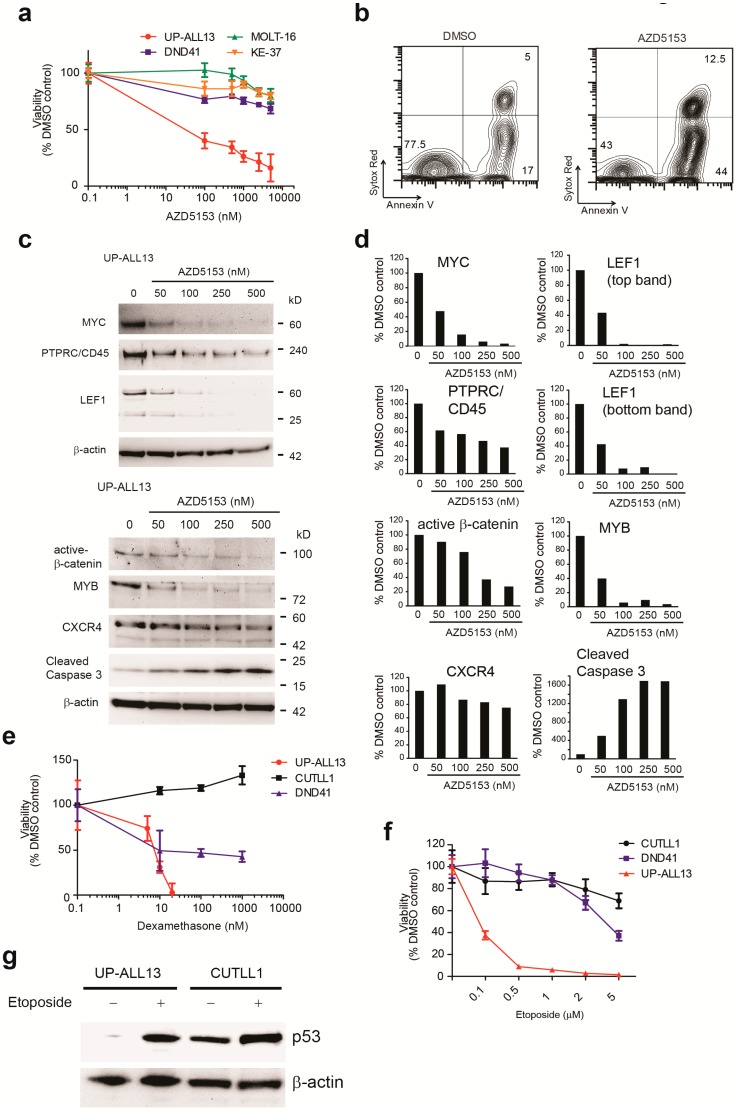
Therapeutic response of UP-ALL13 to the Bromodomain-containing protein 4 (BRD4) inhibitor AZD5153 and other chemotherapeutic drugs. (**a**) Effect of BRD4 inhibition on the viability of UP-ALL13 and the T-ALL cell lines CUTLL1, DND41, MOLT-16, and SKW3/KE-37. Viability was evaluated after 24 h of incubation with increasing doses of AZD5153. (**b**) Representative plots of apoptosis in UP-ALL13 cells treated in vitro with vehicle or AZD5153 (100 nM) for 24 h. (**c**) Western blot analysis of MYC, active β−catenin, CXCR4, CD45/PTPRC, MYB, and LEF1 in UP-ALL13 cells treated in vitro with vehicle or AZD5153 (50–500 nM) for 18 h. Cleaved caspase 3 is used as a marker of apoptosis. β-actin is shown as loading control. (**d**) Quantification of protein loss (from panel **c**) following BRD4 inhibition using AZD5153. The density of each band was quantified by Image J software. Raw signal intensities of each protein were normalized to their loading control (β-actin) and expressed relative to the DMSO-treated control (arbitrarily set to 100). (**e**) Effect of dexamethasone on the viability of UP-ALL13 and the T-ALL cell lines CUTLL1 and DND41. Viability was evaluated after 48 h of incubation with increasing doses of dexamethasone (5 nM–10 μM). (**f**) Effect of etoposide on the viability of UP-ALL13 and the T-ALL cell lines CUTLL1 and DND41. Viability was evaluated after 24 h of incubation with increasing doses of etoposide (100 nM–5 μM). (**g**) Western blot analysis showing the effect of etoposide (1 μM) on the stability of p53 in *TP53* wild-type UP-ALL13 and the *TP53* mutant T-ALL cell line CUTLL1. β-actin is shown as a loading control.

Cell lines are often utilized to screen therapeutic agents relevant to the therapy of the respective malignancy. Given the peculiar genetic properties of our newly established cell line, we evaluated the effectiveness of several different therapeutic agents which could be effective in this model of NOTCH1-independent leukemia. BET chromatin reader proteins, particularly BRD4, play a critical role in many hematological and solid tumors, by acting as coactivators for the expression of proliferative genes. Particularly, BRD4 is required for the expression of MYC, an oncogenic driver in many cancers [35,36]. The genes particularly sensitive to BET inhibition are typically associated with large clusters of enhancers highly enriched with the initiation cofactor Mediator, BRD4, and H3K27Ac, and have been termed as super-enhancers (initially known as locus control regions) [29,37]. Super-enhancers, like enhancers, often upregulate gene expression through long-range interactions with the promoters of those genes [38]. This observation has attracted considerable attention, as therapeutic inhibition of BRD4–histone interactions represent a novel strategy to target MYC-dependent cancers. Since deregulated expression of MYC through *TCRA/D-MYC* translocation (and also possibly through *PTEN* loss [12]) is a trait of this cell line, and in a recently identified NOTCH1-independent/MYC-mediated T-ALL subset [12] we evaluated the efficacy of a new, potent and selective BET/BRD4 bromodomain inhibitor, AZD5153 [39]. This compound, in contrast to JQ1, is a bivalent BET bromodomain inhibitor [39]. As shown in Figure 5a, UP-ALL13 was very sensitive (half maximal inhibitory concentration, IC_50_ < 100 nM at 24 h) to the BET/BRD4 bromodomain inhibitor AZD5153, as compared to other common T-ALL cell lines harboring *NOTCH1*-activating mutations (DND41) or the rare t(7;9) translocation, leading to a constitutively active truncated membrane-bound form of NOTCH1 (CUTLL1; not shown). Surprisingly, other *MYC*-rearranged T-ALL cell lines (MOLT-16, SKW-3/KE-37), while reported to be relatively sensitive to the BET/BRD4 inhibitor JQ1 [8] (and Figure S3), resulted much more resistant to AZD5153 compared to UP-ALL13 at 24 h, much like the non-*MYC* translocated cell line DND41. On the other hand, UP-ALL13 was also highly sensitive to JQ1 (Figure S3). The loss of viability following AZD5153 exposure of UP-ALL13 was mainly due to marked induction of apoptosis (Figure 5b) and not due to cell cycle arrest (data not shown). Western blot analysis of UP-ALL13 cells treated with different doses of AZD5153 demonstrated a dose-dependent decrease in oncogenic drivers putatively under the control of super-enhancers such as MYC, MYB, and β-catenin, which was associated with marked induction of cleaved caspase 3 (Figure 5c,d). Interestingly, other non-transcription factor molecules (PTPRC/CD45 and CXCR4) putatively under the control of super-enhancers showed a less pronounced dose-dependent decrease following exposure to AZD5153 (Figure 5c,d).

In addition to AZD5153, we also evaluated the sensitivity of UP-ALL13 to glucocorticoids (dexamethasone) and etoposide. We found the UP-ALL13 cell line to be exquisitely sensitive to dexamethasone (IC_50_ ≈10 nM at 48 h), especially compared to other common T-ALL cell lines (Figure 5e) such as DND41 (IC_50_ ≈200 nM; glucocorticoid-sensitive) and CUTLL1 (IC_50_ >10 μM; glucocorticoid-resistant).

Sequence analysis of the *TP53* gene in UP-ALL13 did not show the presence of any mutation within its coding region (data not shown), and coherently the UP-ALL13 cell line was found to be highly sensitive to the DNA-damaging agent etoposide, in contrast to p53 mutant T-ALL cell lines such as DND41 and CUTLL1 (Figure 5f). Western blot analysis demonstrated considerable p53 stabilization following etoposide treatment, further corroborating a functional p53 response (Figure 5g) in this cell line, in contrast to the p53 mutant CUTLL1 cell line where p53 stabilization was already present under basal conditions. Overall, these studies demonstrate that UP-ALL13 is a useful tool for testing therapeutic agents targeting signaling pathways de-regulated in T-ALL.

## 4. Discussion

Translocation t(8;14)(q24;q11), initially described in pediatric patients and detected in ≈ 1–6% of T-ALL cases, has been associated with an aggressive disease characterized by hyperleukocytosis, lymphoma-like presentation, rapid neurological progression, and poor response to chemotherapy [7,40]. The main clinical characteristics of the patient from whom the NOTCH1-independent cell line UP-ALL13 was derived fits this description (Table S2). These T-ALLs cluster with TAL1/LMO2-rearranged mature leukemias based on their gene expression signature [7,8]. In t(8;14)(q24;q11), the *MYC* proto-oncogene is over-expressed through its juxtaposition to the *TCR A/D* locus. The genetic profile of these t(8;14)(q24;q11)-translocated T-ALLs is often characterized by *NOTCH1*wt/*FBXW7*wt/*PTEN* mutation or deletion and agrees with that of putative leukemia-initiating cells described in a *Pten*-null mouse T-ALL model.

The UP-ALL13 cell line shares the genetic and immuno-phenotypic characteristics of the parental leukemia cells, including a lack of *NOTCH1* and *TP53* mutations, while maintaining the heterozygous *DNMT3A* R882H mutation. The mutational spectrum of *DNMT3A* seems to be different in (adult) T-ALL cases compared to myeloid malignancies such as AML. Although almost all *DNMT3A* mutations in AML are heterozygous, T-ALL patients frequently harbor homozygous [41] or compound heterozygous *DNMT3A* mutations [42]. Additionally, the distribution of the mutations in *DNMT3A* is more diverse in T-ALL, with the prevalence of mutations affecting the R882 “hot spot” accounting for less than 20%, compared to over 40% in AML [41,43]. Recent studies indicate that the R882 mutant results in a hypomorphic protein [44] that acts as a dominant negative variant, restraining the methyltransferase activity of the remaining wild-type DNMT3A protein. This heterozygous mutation at R882 reduces the methyltransferase activity to approximately 20% of that of the wild-type protein [45,46]. On the other hand, other heterozygous *DNMT3A* mutations (non-R882) can only lower the methyltransferase activity to about 50% of the wild-type protein, suggesting that these mutations may not be sufficient to drive malignancy on their own but select for a second mutation (or loss of heterozygosity) [43]. The prognostic importance of the *DNMT3A* mutations is debated, but on the whole they have no impact on outcome, at least in primary AML [41,43]. In T-ALL, patients with *DNMT3A* mutations have been reported to have a significantly poorer overall survival compared to patients with wild-type DNMT3A [41], but it is not clear whether this finding is just a consequence of the fact that *DNMT3A* mutations are enriched in the more immature T-ALL subtypes, which show a poorer prognosis compared to mature T-ALL [26,42,47]. Interestingly, notwithstanding the presence of the R882 *DNMT3A* mutant in UP-ALL13, we found little evidence of therapeutic efficacy of DOT1L inhibition. This finding suggests that the mechanism of action of *DNMT3A* mutations in T-ALL may be different to AML, possibly due to the necessity of contributing genetic or epigenetic aberrations in T-ALL compared to AML [27].

Another interesting finding was that BRD4 inhibition using AZD5153 resulted particularly effective in contrasting viability and proliferation of UP-ALL13 cells. This supports the idea that this cell line is “addicted” to oncogenes reliant on super-enhancers for high-level expression such as MYC. Particularly intriguing were the results of the H3K27ac ChIP-seq, useful for identifying putative super-enhancers. In fact, putative super-enhancers were associated with transcription factors implicated in: (1) lymphocyte development, including T-lineage specification and maturation (IKZF1, IKZF2, ETS1); (2) T-ALL transcription factor oncogenes (MYB, MYC); (3) Wnt/ß-catenin signaling (LEF1); (4) cell cycle regulators (CCND3); (5) invasion/metastasis genes (CXCR4); and (6) anti-apoptotic members of the Bcl-2 family (BCL2). Interestingly, we found super-enhancers near not only oncogenes but also tumor suppressor genes (*IKZF1*, *LEF1*, *RUNX1*), implying that not all super-enhancer associated genes contribute to tumor progression. Recently, other groups have also reported tumor suppressor gene association with super-enhancers [48,49]. Thus, in some instances the preferential targeting of super-enhancer associated tumor suppressor genes may lead to tumor progression rather than regression.

Altogether, our collective results seem to suggest that certain types of NOTCH1-independent human T-ALL have de-regulated expression of MYC, PTEN inactivation, and possibly β-catenin activation, as recently reported for some *Pten*-deleted mouse models of T-ALL [11,50]. Very recently, H3K27ac ChIP-seq was performed in MOLT-16 cells harboring the t(8;14)(q24;q11) translocation [8]. In this cell line, the highest levels of H3K27ac were found in the enhancer elements of the *TCRA*/*TCRD* locus known to drive *MYC* expression. Consistently, in this study MOLT-16 cells resulted particularly sensitive to the BRD4 inhibitor JQ1 [8] (known to target MYC). We found UP-ALL13 also to be highly sensitive to JQ1 (Figure S3), confirming the therapeutic efficacy of BET bromodomain inhibition in this leukemia model. On the other hand, we found MOLT-16 cells to be more resistant to the bivalent BET bromodomain inhibitor AZD5153, especially at early time points (24 h). This molecule has been found to target a more varied transcriptional/signaling program compared to JQ1 including MYC, E2F, and mTOR [39], suggesting that MOLT-16 cells may not be so reliant on these additional pathways for their proliferation. Altogether our findings suggest that UP-ALL13 cells may represent a more faithful model of transcriptionally addicted cancer cells and could be useful to gain mechanistic insights on the action of BRD4 inhibitors.

In conclusion, we describe a cell line, UP-ALL13, derived from a patient with T-ALL that was NOTCH1-independent and harbored the t(8;14)(q24;q11) translocation, leading to de-regulated expression of MYC. This cell line may be useful as a model system for dissecting the signaling pathways implicated in T-ALL survival and growth independent from NOTCH1 and for the screening of targeted anti-leukemia agents specific for this T-ALL subgroup.

## Figures and Tables

**Table 1:** Immunophenotype of parental leukemia T-cells, xenograft cells, and the UP-ALL13 cell line.

Marker	Primary Leukemia Cells	Xenograft	UP-ALL 13 Cell Line
CD1a	Neg.	Pos (D)	Neg.
CD2	Pos (B)	Pos (B)	Pos (B)
sCD3	Pos (D)	Pos (M)	Pos (M)
CyCD3	Pos (B)	Pos (B)	Pos (B)
CD4	Pos (D)	PP2	Pos (D)
CD8	Pos (D)	PP2	Pos (H)
CD5	Pos (B)	Pos (B)	Pos (B)
CD7	Pos (B)	Pos (H)	Pos (B)
TCRαβ	Neg.	Pos (D)	Pos (M)
TCRγδ	Neg.	Neg.	Neg.
CD19	Neg.	Neg.	Neg.
HLA-DR	Neg.	Neg.	Neg.
CD16	Neg.	Neg.	Neg.
CD56	Neg.	Neg.	Neg.
CD11a	Pos (B)	Pos (B)	Pos (B)
CD11b	nd	Neg.	Neg.
CD11c	nd	Neg.	Neg.
CD13	Neg.	Pos (D)	Pos (D)
CD14	Neg.	Neg.	Neg.
CD15	nd	PP2	Pos (H)
CD33	Neg.	Neg.	Neg.
CD34	Neg.	Pos (D)	Pos (D)
CD38	nd	Pos (B)	Pos (B)
CD44	Pos (B)	Pos (B)	Pos (B)
CD99	Pos (B)	Pos (B)	Pos (B)

Definition of the antigen expression rating is based on the Associazione Italiana Emato-Oncologia Pediatrica-Berlin-Frankfurt-Munster (AIEOP-BMF) consensus guidelines [18]: Pos = positive, Neg = negative, (B) = bright, (M) = medium, (D) = dim, (H) = heterogeneous, PP1 = partially positive 1, PP2 = partially positive 2; nd = not determined; s = surface; Cy = cytoplasmic.

## Supplementary Materials

The following are available online at http://www.mdpi.com/2073-4409/7/10/160/s1. Table S1: Primer sequences used for Sanger sequencing validation. Table S2: Biological and clinical characteristics of primary patient from whom UP-ALL13 was derived. Table S3: DNA fingerprint of UP-ALL13. Figure S1: Effect of gamma secretase inhibitor (DBZ) treatment on UP-ALL13 cells. Figure S2: Sanger sequencing of the regions found mutated by WES for the genes *LRRC74A*, *PCDHB6*, *PMM1*, *ABCA4*, and *SPATA31D3* in the patient leukemia cells, remission material, xenograft cells, and UP-ALL13 cells. Figure S3: Therapeutic response of UP-ALL13 to the BRD4 inhibitor, JQ1.
