# Supplementary material for: A Novel t(8;14)(q24;q11) Rearranged Human Cell Line as a Model for Mechanistic and Drug Discovery Studies of NOTCH1-Independent Human T-Cell Leukemia
Source: Cells. 2018 Oct 9;7(10):160. doi: 10.3390/cells7100160 (PMC6209910; doi:10.3390/cells7100160)
Supplement: Supplementary file 1 [file cells-07-00160-s001.pdf]

Table S1. Primer sequences used for Sanger sequencing validation

Gene Name		Primer Sequence
ABCA4	Fw	TCTACAGGGAGCCAGGATAAA
ABCA4	Rv	TTTCTCAGCAGCTCAATCCA
DNMT3A	Fw	GAGATGATGTCCAACCCTTT
DNMT3A	Rv	AGAAGATTCGGCAGAACTAAG
LRRC74A	Fw	GTCCCCTCTCTCCCTGTCAC
LRRC74A	Rv	GGAGCTCACCTGGATCAGTT
PCDHB6	Fw	CCCTGCAGTCTTTCGAGTTCC
PCDHB6	Rv	CGCCGAACAGACCGAGCTC
PMM1	Fw	CTCAGGCCCCAGGTTCTG
PMM1	Rv	GAGCCCTGTGTTCCTTGG
SPATA31D3	Fw	CCCTAGAAGCTTCCACGAGA
SPATA31D3	Rv	TCACCCCTGAATCATTCCCT

Table S2. Biological and clinical characteristics of primary patient from whom UPALL13 was derived

Sex	Age at diagnosis (years)	WBC count ($x10^9$/L)	Treatment	Relapse	Death (cause)
M	5	121	AIEOP, HR	Yes	Yes (leukemia progression)

WBC, whole blood cell; AIEOP, Associazione Italiana Emato-Oncologia Pediatrica; HR, high risk

Table S3. DNA fingerprint of UP-ALL13

	**Patient leukemia cells**	**Xenograft cells**	**UP-ALL13 cell line**
**T-cell receptor rearrangement**			
	Vγ2Jγ2.3/Vβ29Jβ2.7	Vγ2Jγ2.3/Vβ29Jβ2.7	Vγ2Jγ2.3/Vβ29Jβ2.7
**STR locus***			
**D3S1358**	14	14	14
**TH01**	9.3	9.3	9.3
**D21S11**	29-30	29-30	29-30
**D18S51**	14-15	14-15	14-15
**Penta E 7-9**	5-12	5-12	5-12
**D5S818**	12-13	12-13	12-13
**D13S317**	8-12	8-12	8-12
**D7S820**	9-13	9-13	9-13
**D16S539**	12	12	12
**CSF1PO**	11-13	11-13	11-13
**Penta D**	9-14	9-14	9-14
**AMEL**	X-Y	X-Y	X-Y
**vWA**	18-20	18-20	18-20
**D8S1179**	14-15	14-15	14-15
**TPOX**	8	8	8
**FGA**	23-24	23-24	23-24
**D19S433**	13-14	13-14	nd
**D2S1338**	17-19	17-19	nd

*Allele nomenclature is shown ; nd=not determined

Figure S1

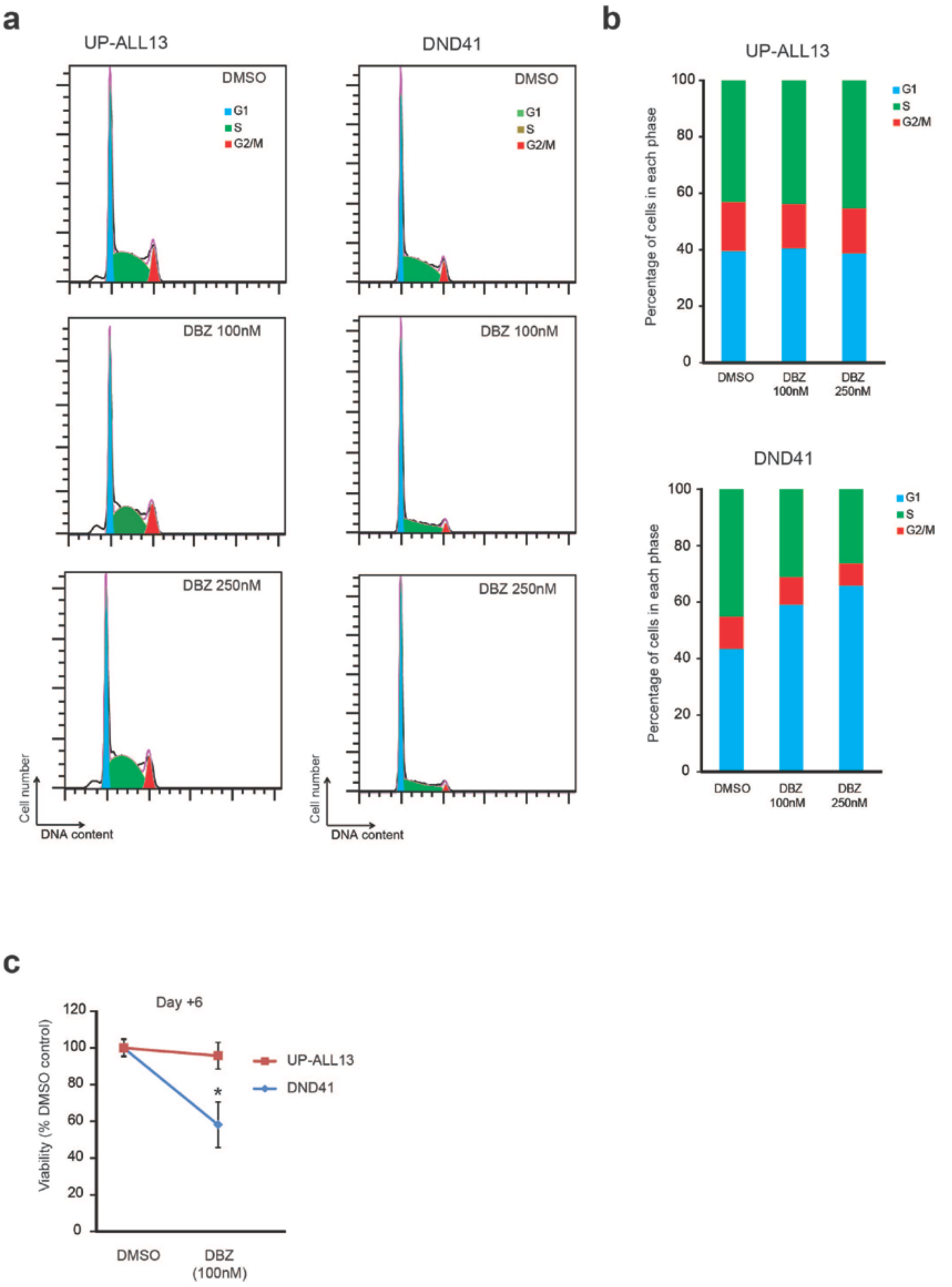

Figure S1. Effect of gamma secretase inhibitor (DBZ) treatment on UP-ALL13 cells. (**a**) Representative cell cycle plots and (**b**) cell cycle phase distribution of UP-ALL13 and DND41 cell lines treated for 6 days with different doses of DBZ (100 and 250 nM). (c) Viability of UP-ALL13 and DND41 cell lines was evaluated after 6 days of incubation with 100 nM of DBZ. Error bars represent ± standard deviation of quadruplicate wells. *, $P< 0.01$.

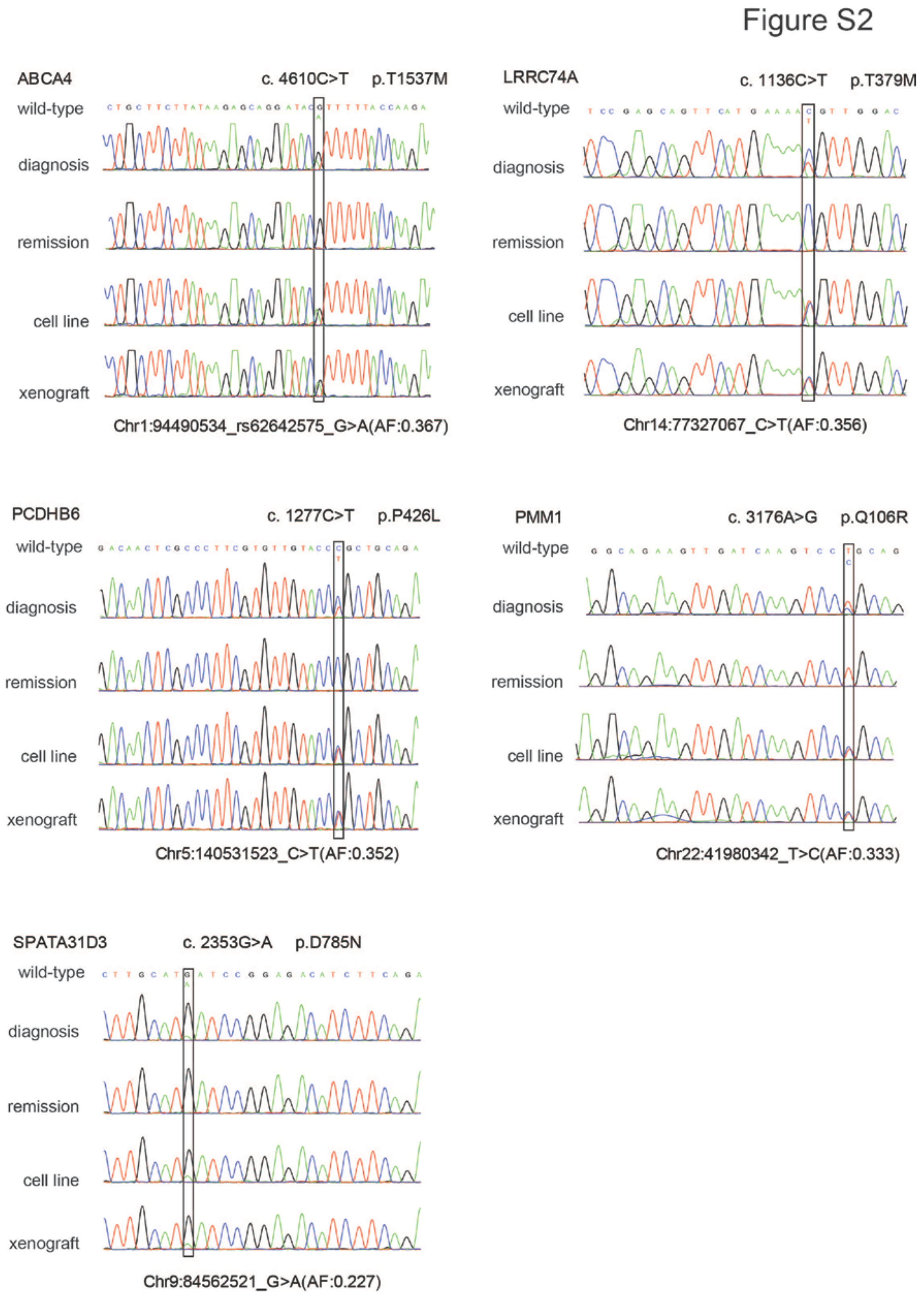

Figure S2. Validation by Sanger sequencing of the regions found mutated by WES for the genes *LRRC74A, PCDHB6*, *PMM1, ABCA4, SPATA31D3* in the patient leukemia cells, remission material, xenograft cells, and UP-ALL13 cells.

Figure S3

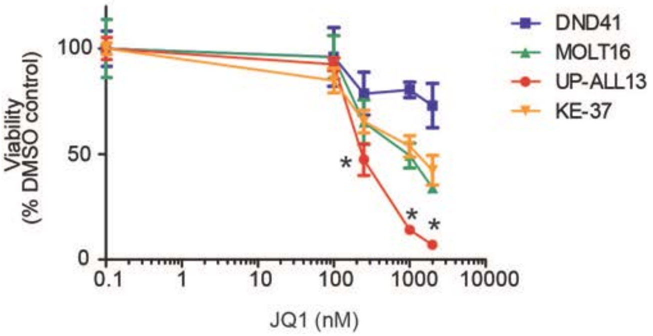

Figure S3. Therapeutic response of UP-ALL13 to the BRD4 inhibitor , JQ1. Effect of BRD4 inhibition on the viability of UP-ALL13 and the T-ALL cell lines : DND41, MOLT-16 and SKW3/KE-37. Viability was evaluated after 48h of incubation with increasing doses of JQ1. Viability is shown as a percentage of the DMSO control treated cells. Error bars represent ± standard deviation of quadruplicate wells. *, $P < 0.01$.
